# The Association of Electronic Cigarette Use and the Subjective Domains of Physical and Mental Health: The Behavioral Risk Factor Surveillance System Survey

**DOI:** 10.7759/cureus.7088

**Published:** 2020-02-24

**Authors:** Mahmoud Al Rifai, Mohammadhassan Mirbolouk, Olufunmilayo H Obisesan, Xiaoming Jia, Khurram Nasir, Anwara T Merchant, Michael Blaha, Salim Virani

**Affiliations:** 1 Cardiology, Baylor College of Medicine, Houston, USA; 2 Internal Medicine, Yale School of Medicine, New Haven, USA; 3 Epidemiology and Public Health, The Johns Hopkins Ciccarone Center for the Prevention of Heart Disease, Baltimore, USA; 4 Cardiology, Debakey Heart & Vascular Center, Houston Methodist Hospital, Houston, USA; 5 Epidemiology and Biostatistics, Arnold School of Public Health, Columbia, USA; 6 Cardiology, Johns Hopkins Hospital, Baltimore, USA; 7 Cardiology, Michael E. Debakey Veterans Affairs Medical Center and Baylor College of Medicine, Houston, USA

**Keywords:** electronic cigarettes, mental health, physical health

## Abstract

Introduction

Individuals who use electronic cigarettes (e-cigarettes) may have a poor perception of physical and mental health. We, therefore, studied the association of e-cigarettes with subjectively reported health domains.

Methods

We utilized cross-sectional data from the 2016 and 2017 Behavioral Risk Factor Surveillance System (BRFSS), a nationally representative U.S. telephone-based survey. All variables were self-reported. E-cigarette use was characterized as never, former, or current. Health domains included overall health, physical health, and mental health, which was assessed as the frequency of emotional support, life satisfaction, and feeling stressed. Weighted multivariable-adjusted logistic regression models were used to examine the association between e-cigarette use and these health domains.

Results

Our study population consisted of 755,355 (79%) never e-cigarette users, 111,940 (16%) former users, and 28,917 (5%) current users. There was a significant association between e-cigarette use and the less favorable perceived state of overall health, physical health, or mental health. For example, the OR (95% CI) for the association of current e-cigarette use with health domains was as follows: good perception of overall health: 0.78 (0.74,0.83), physical health: 0.69 (0.66,0.73), adequate emotional support: 0.89 (0.79,0.99), feeling satisfied: 0.83 (0.71,0.96), or being free of stress: 0.64 (0.53,0.76). Similar results were obtained in younger individuals (aged 18-34 years).

Conclusions

E-cigarette use is associated with a less favorable perception of physical and mental health as compared to never use, particularly among younger adults. These results have important implications for clinicians for patient counseling and regulatory agencies to regulate e-cigarette sales.

## Introduction

Electronic cigarettes (e-cigarettes) were introduced in the U.S. about a decade ago and their share of the overall tobacco product market has risen exponentially. They are marketed as a less harmful alternative to conventional cigarette smoking and as an aid for quitting or reducing cigarette smoking [1]. Such claims are yet to be substantiated in the absence of long-term follow-up data. The increasing appeal of e-cigarettes to younger adults is concerning, given that e-cigarettes may act as a gateway to cigarette smoking [2]. In particular, individuals with a poor perception of health may be more susceptible to using e-cigarettes [3-4]. Currently, the association between e-cigarette use and the perceptions of physical and mental health is unknown.

We, therefore, sought to study the association of e-cigarette use with subjectively reported health domains. This may help understand physical and psychosocial factors related to e-cigarette use and provide guidance to tobacco regulatory agencies for marketing e-cigarettes.

## Materials and methods

The Behavioral Risk Factor Surveillance System (BRFSS) survey, established by the Centers for Disease Control and Prevention, is a nationwide telephone-based questionnaire of a random sample of U.S. residents regarding health-related risk behaviors, chronic health conditions, and the use of preventive services. BRFSS includes participants in all 50 states as well as the District of Columbia and three U.S. territories, making it the largest telephone-based survey in the world. We utilized data from the 2016 and 2017 BRFSS survey. As BRFSS is a deidentified dataset that is publicly available (http://www.cdc.gov/brfss), it did not require institutional review board (IRB) approval.

E-cigarette status was characterized as never vs. ever based on the participants’ response to the question “Have you ever used an e-cigarette or other electronic vaping product, even just one time, in your entire life?”. Ever users were then classified as current vs. former depending on their answer to the questions “Do you now use e-cigarettes or other vaping products every day, some days, or not at all?” Subjectively reported physical and mental health were defined based on the participants’ answers to questions pertaining to health domains (Table 1) and coded as binary.

**Table 1:** Multivariable-adjusted odds ratios (95% confidence interval) for the association between e-cigarette use and physical and mental health *Stress refers to a situation in which a person feels tense, restless, nervous, anxious, or is unable to sleep at night because his/her mind is troubled all the time ** Never e-cigarette users are selected as the reference category i.e. odd ratio = 1 φ Model is adjusted for age, gender, race/ethnicity poverty level, education, heavy alcohol use, employment status, marital status, and cigarette smoking status

Mental health condition	Question asked	Health factor definition	Never e-cigarette users**	Former e-cigarette users		Current e-cigarette users	
				Unadjusted	Adjusted	Unadjusted	Adjusted
Perception of overall health	Would you say that in general your health is?	“Very good or excellent”	1 (ref)	0.85 (0.83,0.87)	0.88 (0.85,0.91)	0.74 (0.70,0.77)	0.78 (0.74,0.83)
Physical health	Number of days physical health is not good?	“0”	1 (ref)	0.77 (0.75.0.79)	0.78 (0.76,0.81)	0.66 (0.63,0.69)	0.69 (0.66,0.73)
Emotional support	How often do you get the social and emotional support you need?	“Always”	1 (ref)	0.80 (0.75,0.85)	0.79 (0.73,0.87)	0.84 (0.74,0.95)	0.83 (0.71,0.96)
Feeling stressed	Within the last 30 days, how often have you felt stress*?	“None of the time”	1 (ref)	0.56 (0.52,0.60)	0.65 (0.59,0.72)	0.59 (0.51,0.67)	0.64 (0.53,0.76)
Life satisfaction	In general, how satisfied are you with your life?	“Satisfied or very satisfied”	1 (ref)	0.84 (0.74,0.95)	0.86 (0.80,0.92)	0.95 (0.87,1.05)	0.89 (0.79,0.99)

We analyzed these cross-sectional data using survey weights as the BRFSS uses design weighting and iterative proportional fitting to ensure the representativeness of the data to the US population [5]. Baseline characteristics, including demographics, substance use, and subjective physical and mental health, were tabulated by e-cigarette use status (never, former, current). To study the association of e-cigarette use with subjective health domains, we used weighted multivariate logistic regression models adjusting for age, gender, race/ethnicity poverty level, education, heavy alcohol use, employment status, marital status, and cigarette smoking. In these analyses, never users of e-cigarettes were selected as the reference category (odds ratio 1). We also conducted subgroup analyses by age, gender, race/ethnicity, and cigarette smoking status. A p-value of <0.05 was considered statistically significant. All analyses were conducted using Stata version 13.1 (StataCorp, College Station, Texas).

## Results

Our study population consisted of 755,355 (79%) never e-cigarette users, 111,940 (16%) former users, and 28,917 (5%) current users. The prevalence of current smoking was 16%. Compared to never users, current or former e-cigarette users were younger, with ~50% between the ages of 18 and 34 years, more likely to be men, single, employed, and current cigarette smokers, but less likely to have access to a primary care physician. They also demonstrated a less favorable perception of physical or mental health and were more likely to report feeling stressed (all p <0.05).

In multivariable analyses, former and current e-cigarette use were associated with a less favorable perception of health in various domains compared to never users. For example, the OR (95% CI) for the association of current e-cigarette use with health domains were as follows: good perception of overall health: 0.78 (0.74,0.83), physical health: 0.69 (0.66,0.73), adequate emotional support: 0.89 (0.79,0.99), feeling satisfied: 0.83 (0.71,0.96), or being free of stress: 0.64 (0.53,0.76) (Table 1). Similar results were obtained in different age groups, particularly among those aged 25-34 years. There were also no significant differences by gender, race or ethnicity, cigarette smoking status, access to primary care physician, employment, or marital status. Further adjustment for comorbidities (hypertension, diabetes, chronic kidney disease, hypercholesterolemia, history of myocardial infarction, history of stroke, and chronic obstructive pulmonary disease) also yielded similar findings (results not shown).

## Discussion

In a nationally representative sample, e-cigarette use was associated with less favorable perceived physical and mental health as compared to never use.

Few studies have evaluated the association of e-cigarettes and the perception of physical and mental health. In a U.S. population survey, Cummins et al. found that individuals with mental illness were more likely to have tried e-cigarettes as compared to those without such conditions [6]. Our results from a nationally representative sample of U.S. adults indicate that ever e-cigarette use is associated with a perception of poor physical or mental health indicators. Therefore, an assessment of e-cigarettes may be an important consideration when evaluating individuals with a poor perception of physical and mental health. For example, perceived poor physical health or lack of emotional support or feeling stressed may lead some individuals to use e-cigarettes. Alternatively, individuals using e-cigarettes may feel stigmatized by family or friends and, therefore, report poorer health. Clinicians evaluating physical and mental health factors that may be underlying e-cigarette use may help result in cessation in current users or prevention of relapse in former users. The results of this study can also be used by government agencies, such as the Food and Drug Administration, to regulate the marketing or sale of e-cigarettes to vulnerable individuals such as younger adults who may be prone to using e-cigarettes due to poor perceived physical or mental health.

Our results must be interpreted in the context of important limitations. This was a cross-sectional study and, therefore, causality cannot be inferred. In an epidemiologic study, there is the possibility of residual confounding. As information was self-reported, it is subject to measurement error and response bias.

## Conclusions

In conclusion, e-cigarette use appears to be associated with an adverse perception of physical and mental health, particularly among younger adults. E-cigarette use is associated with a poor perception of overall health or physical health, poor emotional support, dissatisfaction, and a feeling of stress. Further studies are required to assess whether interventions aimed at improving physical or mental health may prevent e-cigarette use or help e-cigarette users quit the habit.
